# Correlating Intravital Multi-Photon Microscopy to 3D Electron Microscopy of Invading Tumor Cells Using Anatomical Reference Points

**DOI:** 10.1371/journal.pone.0114448

**Published:** 2014-12-05

**Authors:** Matthia A. Karreman, Luc Mercier, Nicole L. Schieber, Tsukasa Shibue, Yannick Schwab, Jacky G. Goetz

**Affiliations:** 1 European Molecular Biology Laboratory Heidelberg, Heidelberg, 69117, Germany; 2 Inserm U1109, MN3T, Strasbourg, F-67200, France; 3 Université de Strasbourg, Strasbourg, F-67000, France; 4 LabEx Medalis, Université de Strasbourg, Strasbourg, F-67000, France; 5 Fédération de Médecine Translationnelle de Strasbourg (FMTS), Strasbourg, F-67000, France; 6 Whitehead Institute for Biomedical Research, Massachusetts Institute of Technology, Cambridge, Massachusetts, United States of America; Glasgow University, United Kingdom

## Abstract

Correlative microscopy combines the advantages of both light and electron microscopy to enable imaging of rare and transient events at high resolution. Performing correlative microscopy in complex and bulky samples such as an entire living organism is a time-consuming and error-prone task. Here, we investigate correlative methods that rely on the use of artificial and endogenous structural features of the sample as reference points for correlating intravital fluorescence microscopy and electron microscopy. To investigate tumor cell behavior *in vivo* with ultrastructural accuracy, a reliable approach is needed to retrieve single tumor cells imaged deep within the tissue. For this purpose, fluorescently labeled tumor cells were subcutaneously injected into a mouse ear and imaged using two-photon-excitation microscopy. Using near-infrared branding, the position of the imaged area within the sample was labeled at the skin level, allowing for its precise recollection. Following sample preparation for electron microscopy, concerted usage of the artificial branding and anatomical landmarks enables targeting and approaching the cells of interest while serial sectioning through the specimen. We describe here three procedures showing how three-dimensional (3D) mapping of structural features in the tissue can be exploited to accurately correlate between the two imaging modalities, without having to rely on the use of artificially introduced markers of the region of interest. The methods employed here facilitate the link between intravital and nanoscale imaging of invasive tumor cells, enabling correlating function to structure in the study of tumor invasion and metastasis.

## Introduction

Electron Microscopy (EM) uniquely enables imaging the object of interest at high resolution in its structural context. In the study of metastatic processes, EM is the only technique to reveal the ultrastructure of both the invading cell and its microenvironment. When targeting rare and transient events like invasion, however, the small field of view of the EM and its restriction to image immobilized specimens are a disadvantage. *In vivo* studies of metastatic tumor cells have been successfully performed previously by intravital two-photon excitation microscopy (2PEM) of fluorescent tumor cells [1]. The infrared light used in 2PEM penetrates deep into the sample, and the effects of photo-bleaching and phototoxicity are reduced overall [2]. Moreover, using a single excitation wavelength, it is possible to simultaneously image fluorescent dyes, genetically expressed fluorescent proteins, and second or third harmonic signal generated by other features in the tissue like collagen fibers [3] and lipids [4]. Correlative light and electron microscopy (CLEM), exploits the advantages of both light microscopy and EM in the study of a single sample. When studying metastasis, 2PEM can monitor fluorescent tumor cells migrating through living tissue and image the process of invasion over time. Following fixation and processing, EM imaging then provides a snapshot of the fine structure and microenvironment of the tumor cells.

The main hurdle in CLEM is to keep track of the region of interest (ROI) while moving from the fluorescence to the electron microscope. Various procedures have been developed for the accurate retracing of the ROI in 2D organized samples. Registering the position of the ROI within a coordinate system of reference points can yield a precision of 100 nm [5]–[7]. Alternatively, by storing its xy-coordinates relative to the sample stage the correlation procedure can be partly automated [8]–[11]. However, in bulky and complex samples, like multicellular organisms and tissues, the retrieval of a rare event remains a challenge. Approaches were developed to mark the position of the ROI by Near Infrared Branding (NIRB) of the tissue [12]–[14], or by laser etching of the resin embedded sample [15]. The laser, however, looses power upon deep penetration into the sample, due to scattering. On brain tissue slices, precise marking of the ROI within the tissue was limited to a depth of 50 µm or less [12]–[14]. For larger specimens, it is thus only possible to create marks at the surface of the sample. These markings therefore reveal the xy-coordinates of the ROI with high precision, but fail to provide its z-position within a large 3D specimen. To accurately retrieve the ROI in three dimensions, these methods thus still require browsing through numerous z-sections of the sample. The ROI can then be recognized based on its position relative to structural features in the sample [16], often in conjunction with overlapping the outline of the EM structure with the shape of the fluorescent signal [17]. To help recognizing the cell or structure of interest, it is possible to use bimodal [18], [19] or photo-convertible [20], [21] probes that can be recognized first in FM and next in EM. Although facilitating its identification, the use of these markers can mask the ultrastructure of the ROI [18], result in unspecific labeling [22] or may influence cellular processes [23]. To circumvent this, we set to evaluate different approaches that enable retrieval of the structure of interest imaged deep within a tissue without marking it with artificially introduced labels.

In this work, we employ CLEM to study early events of tumor invasion in mouse in order to correlate tumor cell behavior *in vivo* to their ultrastructural architecture in 3D. Metastasis formation is a multi-step process where tumor cells invade the surrounding tissue of a primary tumor, intravasate into the blood circulation, disseminate to anatomically distant organs and establish themselves in a new tissue microenvironment [24]. The complex bio-mechano-chemical processes leading to these events involve, amongst others, the formation of cellular protrusions, the turnover of cell-matrix contacts and local remodeling and degrading of the extra-cellular matrix surrounding the cells [25]–[28]. Previously, *in vitro* models have provided relevant information on the behavior of invading tumor cells, but there is currently no *in vivo* model system that can combine functional imaging of tumor cells in their microenvironment with their visualization in EM. 2PEM imaging can be performed directly on skin [29] or through an imaging window installed above the organ of interest [30]–[33]. Intravital fluorescence microscopy, however, does not provide detailed insight in the architecture of the imaged areas, stressing the missing link between the dynamics and the ultrastructure of metastatic events.

To enable correlating intravital 2PEM to 3D-EM of invading tumor cells *in situ*, we have used an approach that exploits both artificial and endogenous landmarks visible in the sample. Fluorescent tumor cells are injected in the mouse ear and imaged a few days later by 2PEM. The recording site is then marked by NIRB, at the level of the skin surface, so that the ROI could be retrieved and processed for EM. The ROI is then approached by ultramicrotomy. In this work, we make use of three variants of targeting using the reconstruction of the tissue anatomy from serial sections of the tissue embedded in the resin block. We show how we have made optimal use of tissue features, which are visible in each imaging modality, to achieve accurate correlation. Using the workflow we describe here, it was possible to successfully retrieve tumor cells injected in mouse ear, which enabled us to move from intravital dynamic 2PEM imaging to high-resolution 3D TEM tomography.

## Methods

### Mice handling

**Figure 1:**
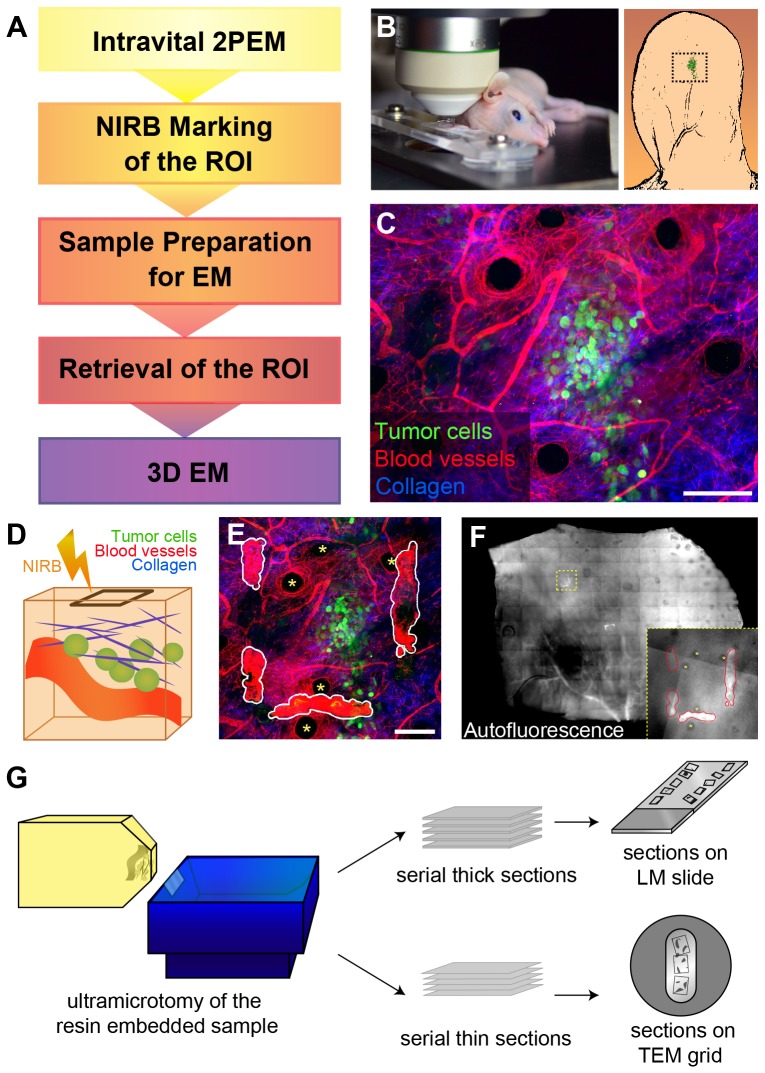
Outline of the Correlative Workflow. A. The flowchart lists the general steps involved in the correlative workflow. B. Left panel: installation of the anesthetized mouse on the LM stage. The ear with the transplanted fluorescent tumor cells (right panel, cartoon) is mounted in a custom-built holder. C. Z-projection of a typical 2PEM dataset obtained from the mouse ear that was injected with GFP-expressing tumor cells (green). SHG signal of the collagen fibers is shown in blue. Evans Blue stains blood vessels and is depicted in red. Scale bar: 100 µm. See Movie S1. D. Cartoon of the NIRB process representing the imaged volume containing a vessel (red), collagen fibers (blue), and tumor cells (green). Our NIRB-procedure entails drawing a frame at the surface of the skin with a high-powered laser, above and away from the ROI. The imaged volumes presented in this work were in between 60 and 200 µm in depth and ranging from 270 to 440 µm in xy. E. Following NIRB, the same volume is imaged again with 2PEM. The NIRB marks visible in this z-projection are traced in white. Scale bar: 100 µm. Asterisks point to hair follicles. See Movie S1. F. After chemical fixation of the mouse ear sample, NIRB markings remain temporarily autofluorescent and their location can be mapped. Asterisks pointing to hair follicles (as in E). G. Following EM processing, the embedded sample can be trimmed and sectioned by ultramicrotomy. Serial thick sections (500 nm) are placed on glass slides to be imaged by light microscopy (‘sections on LM slide’). Serial thin sections (60 to 240 nm) are mounted on slot grids (‘sections on TEM grid’) allowing for TEM observation and/or electron tomography.

**Figure 2:**
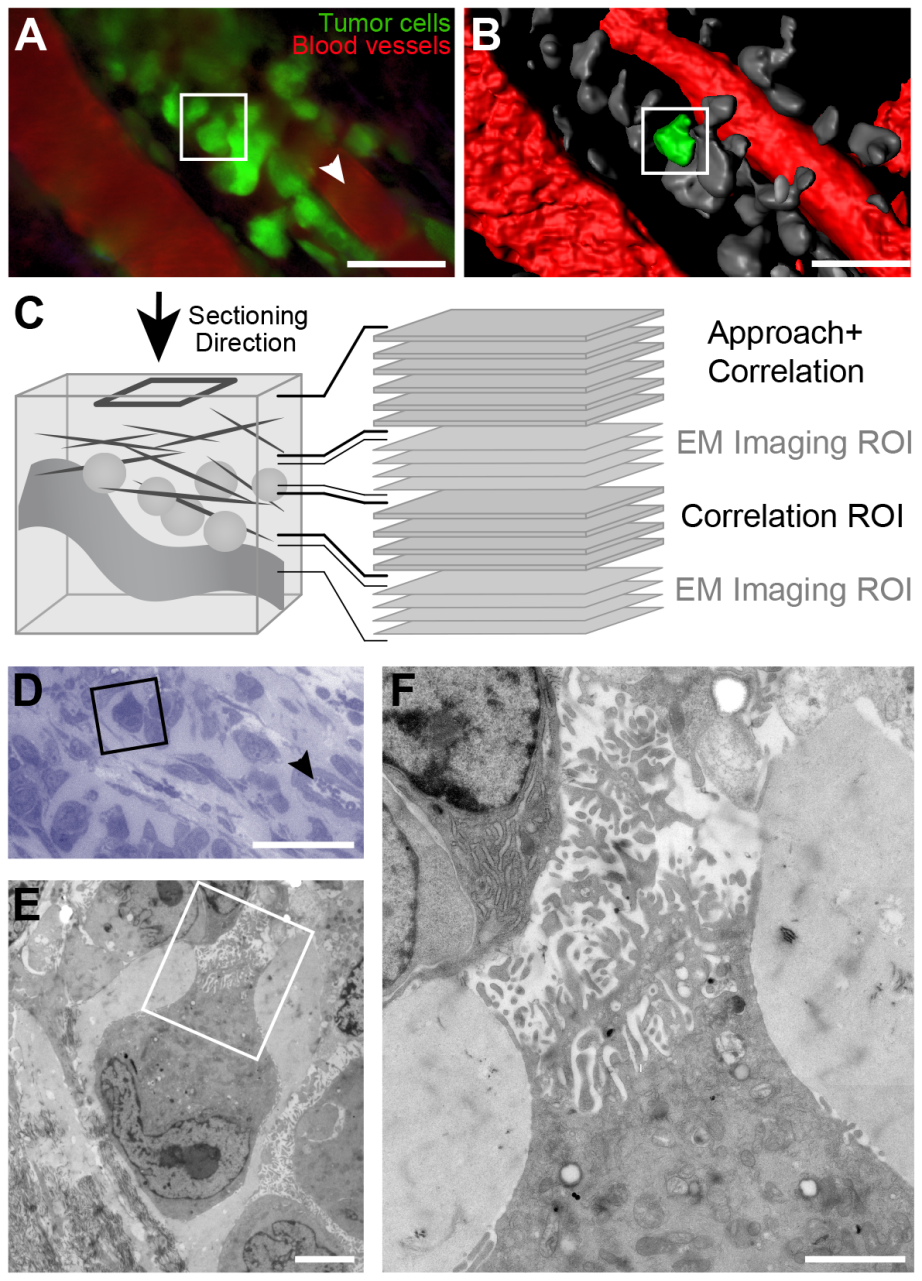
Correlation based on Sequential LM and EM imaging to Target a Single Tumor Cell. A. 3D view of a 2PEM z-stack of mouse ear skin tissue, 7 days post-injection with GFP-expressing tumor cells (green). Vessels are stained with Evans Blue (red). The arrowhead indicates the part of the blood vessel that is also visible in the thick section shown in panel D. The image is obtained using the 3D Viewer plugin in Fiji. Scale bar: 50 µm. See Movie S2. B. 3D model of the imaged volume. The cell of interest, boxed in panel A and B, is segmented in green, the other cells are segmented in grey. The vessels are shown in red. Scale bar: 50 µm. C. Cartoon of the sectioning procedure. The resin embedded sample is sectioned from the direction indicated with the arrow. To approach the ROI, 180 thick sections (500 nm) were obtained from the sample ('Approach and Correlation'). Next, 10 consecutive series of 10 thin (60 nm) and two thick (500 nm) sections were obtained from the ROI. Thick sections were used for correlation and the thin sections were imaged with EM. D. The cell of interest (box) was identified in a thick toluidine blue stained section of the ROI. The arrowhead points to a cross-section of the vessel depicted in A with a white arrowhead. Scale bar: 50 µm. E: Low magnification EM image of the cell boxed in panel D. The cell appears highly polarized and contains protrusive structures, showed at higher magnification in panel F. Scale bar in E: 5 µm, in F: 2 µm. See Movie S2.

**Figure 3:**
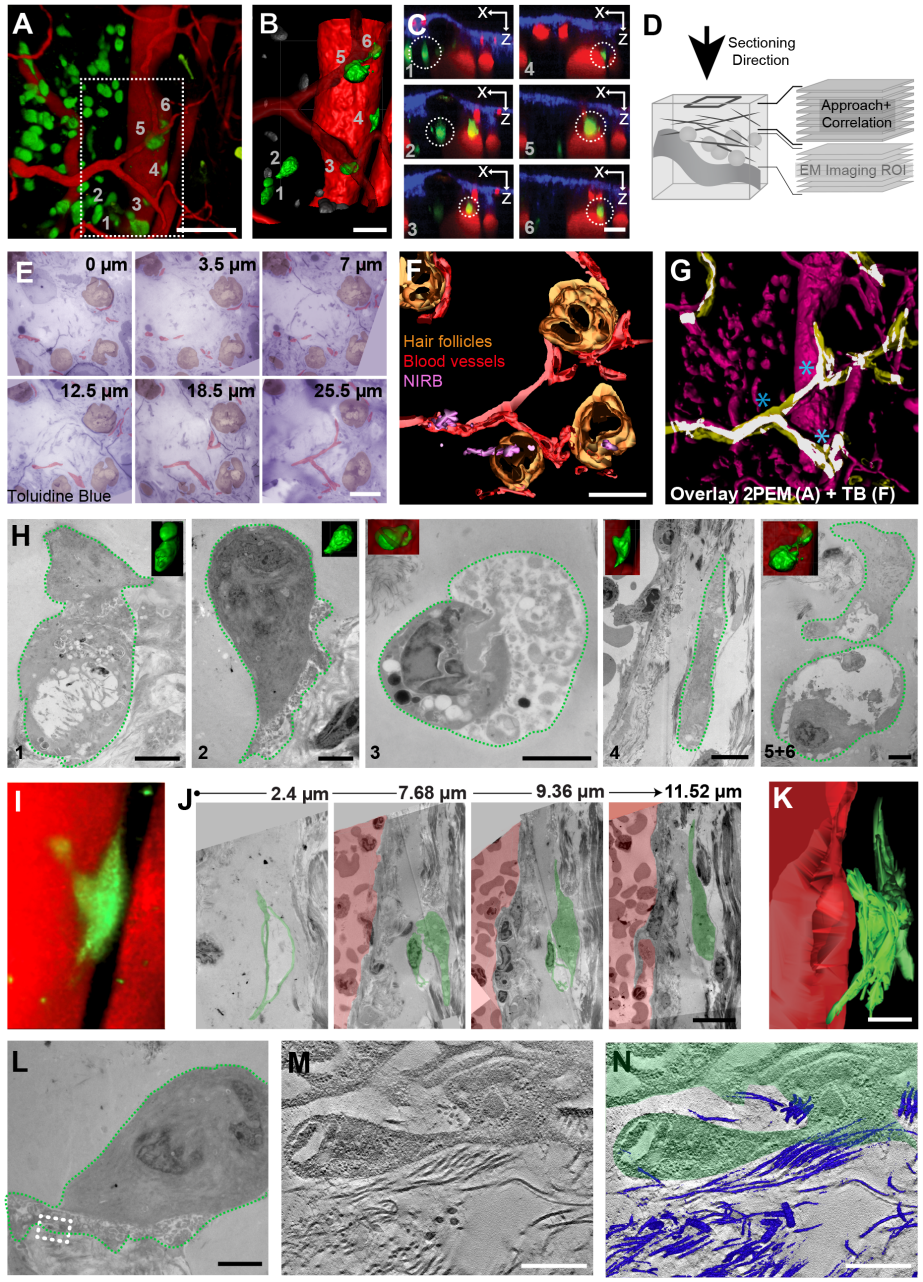
Full Volume Correlation of the ROI. A. 3D view of a 2PEM z-stack of mouse ear tissue, 7 days post-injection with GFP-expressing tumor cells (green). Vessels are stained with Evans Blue (red). Scale bar: 100 µm. See Movie S3. B. 3D model of the area that is indicated with a box in A. The cells of interest are segmented in green and numbered (1–6). The vessels are shown in red. The vessel bifurcation close to the skin surface is made transparent to reveal the cells underneath. Scale bar: 50 µm. C. X-Z slices through the 3D 2PEM volume, at the height of the cells of interest (circled). The numbers on the left bottom correspond to the numbers used in panel B to label the cells of interest. These views show the relative position of the cells with respect to the blood vessels (red) and the collagen-rich dermis (blue). D. Cartoon of the sectioning procedure. To approach the ROI and monitor the progression, serial thick sections were produced from the sample (the arrow indicates the sectioning direction) and imaged with LM (‘Approach+Correlation’). Approximating the location of the cells of interest, 240 nm sections for electron tomography were obtained (‘EM imaging ROI’). E. 500 nm toluidine-blue-stained sections that were obtained at different depths within the sample (the relative z-distance from the first section is indicated in top right corner of each panel). The vessels are segmented in red, hair follicles in brown. Scale bar: 100 µm. See Movie S3. F. 3D model of the hair follicles (brown), vessels (red) and NIRB markings (purple) segmented in the serial thick sections. Scale bar: 50 µm. See Movie S3. G. Overlay of a 3D model of the 2PEM z-stack (based on A, magenta) and a model of the vessels segmented in the serial thick sections (based on F, yellow). Overlapping areas are highlighted in white. Asterisks indicate vessel-forks that are visible in both datasets. Scale bar: 50 µm. See Movie S3. H. Correlation between 2PEM, LM and EM enabled retrieving the cells of interest (numbering in bottom right corner, as in B and C) in the sequence of serial thin sections. The electron transparent areas in proximity of the cells indicate a local absence of extracellular matrix components. The cause of this missing material remains to be elucidated. I. A selected area in the 2PEM dataset, showing a cell of interest (number 4 in B, C and H). J. Selection of the TEM images of cell 4, shown in panel I. The cell is modeled in green and the vessel lumen in red. For each image, we indicated the z-distance from the section where the cells was first spotted. Scale bar: 10 µm. K. Cell 4 (green) and the blood vessel lumen (red) are segmented in the serial sections (J). A 3D model of this segmentation reveals the shape of the cell, which closely resembles the 2PEM view of the same cell, shown in I. Scale bar: 10 µm. L-N: Electron tomography of a tumor cell (number 2 in B,C, and H), shown at low magnification in panel L. At the position of the box in L, a tomogram was obtained. Scale bar: 5 µm. See Movie S3. M. One selected virtual slice of the tomogram shows the complex architecture of the cell membrane and the fiber-like structure of extracellular matrix (ECM). N: The high image contrast of the ECM fibers enables semi-automatic segmentation of the fibers (shown in blue) within the 3D dataset. Scale bars in M and N: 500 nm. See Movie S3.

**Figure 4:**
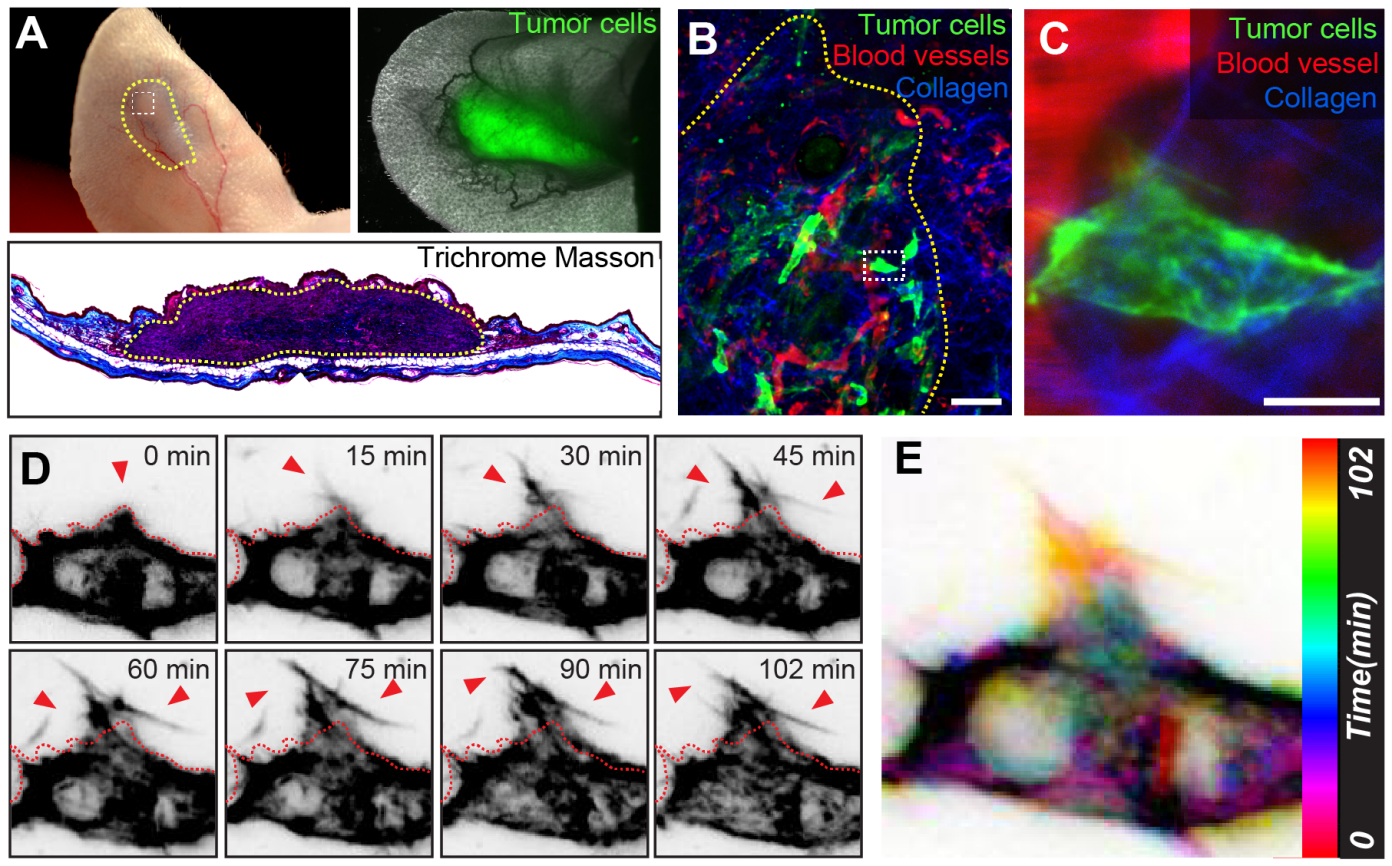
2PEM Imaging of D2.0R Cells Xenotransplanted in Mouse Ear Skin. A. Two weeks after injection with LifeAct-Ypet expressing D2.0R cells, a tumor is formed in the mouse ear skin (top left panel, outlined in green). The green fluorescent signal of the D2.0R cells can be observed in the tumor region (top right panel). A histological cross-section through the tumor region was obtained from the second ear injected at the same time with the same cell type. The Trichrome Masson staining colors the collagen in blue (bottom panel). B. Maximum projection of a z-stack obtained from an area in the periphery of the invasive tumor (top left panel A, white square). The D2.0R cells are shown in green, and the tumor is outlined in yellow. A background signal from the Evans Blue (red) can be observed in the vicinity of the blood vessels, potentially caused by the ‘leaky’ nature of the tumor vessels, its uptake by residing immune cells and the repeated injections. Scale bar: 50 µm. See Movie S4. C. Higher magnification z-projection of the cell of interest boxed in B. Scale bar: 10 µm. D. Time-lapse imaging of a D2.0R tumor cell in vivo. The different panels depict, in black against a white background, the fluorescent signal of the actin in the cell at the indicated time-points. See Movie S4. E. Color-coded map of the structural changes of the cell of interest (boxed in B and C) over time. Scale bar: 10 µm.

**Figure 5:**
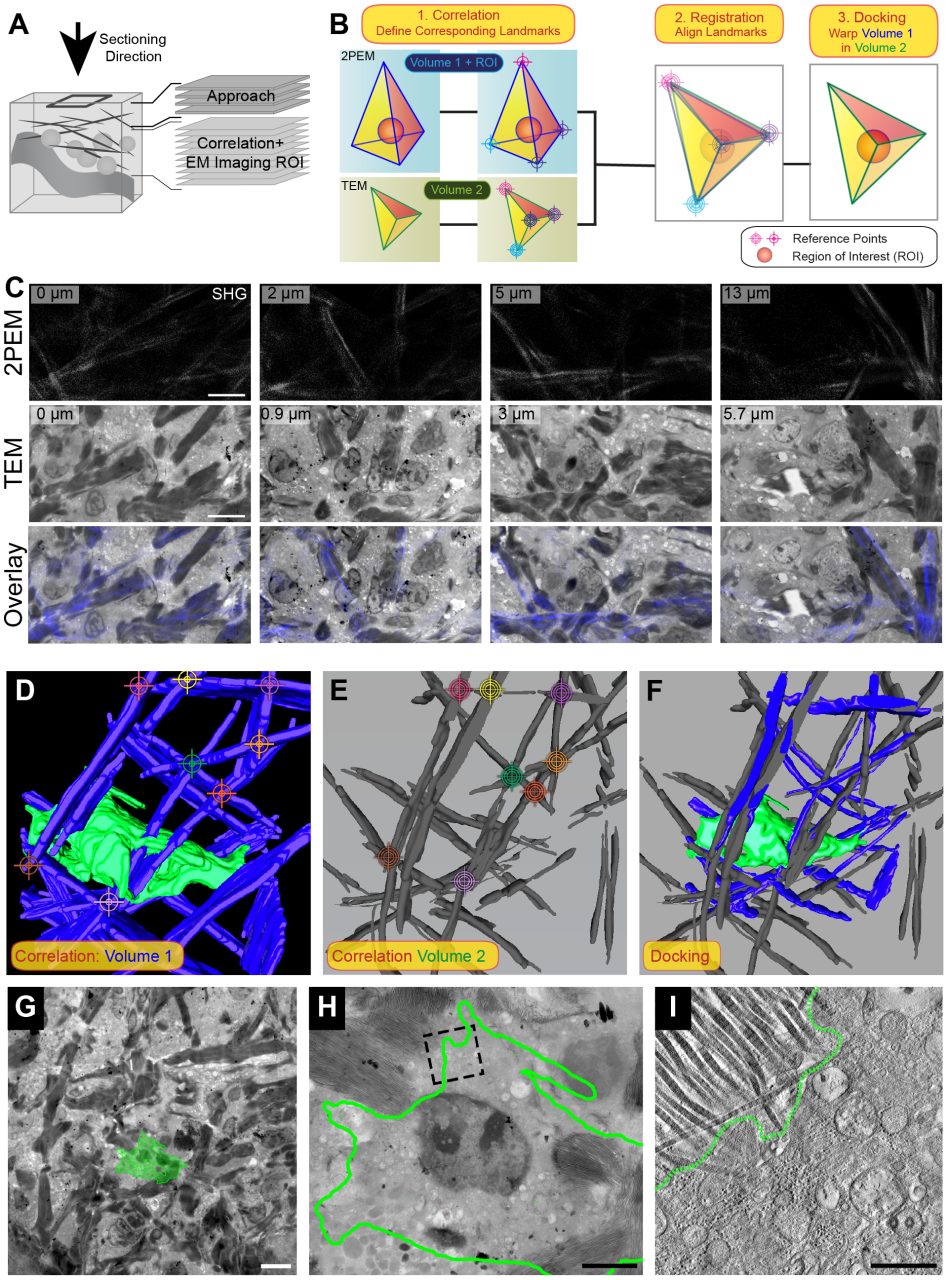
Correlating 2PEM to 3D EM of D2.0R Cells using Collagen Fibers as a Reference. A. Cartoon of the sectioning approach. Following 2PEM, NIRB and processing as described before, a few thick sections were produced from the sample to verify the orientation of the block and the presence of the NIRB markings (‘Approach’). The sectioning direction is indicated with an arrow. As soon as the first NIRB marks were spotted in the thick sections, we proceeded with obtaining thin 240 nm sections for electron tomography (‘Correlation+EM imaging ROI’). These sections were not only employed for EM imaging of the ROI, but also provided the structural features that were used for correlation between 2PEM and EM. B. Graphic representation of the landmark-based correlation procedure. The prisms with the blue and green ribs represent similar 3D volumes that differ in size, shape and orientation, representing the samples pre- and post-EM-processing. In order to predict the position of the ROI (ball, visible in volume 1) in volume 2, similar points (landmarks) in both datasets are identified (‘Correlation’). These landmarks are overlaid in 3D (‘Registration’) enabling warping of volume 1 into volume 2 and thus also projecting the position of the ROI in volume 2 (‘Docking’). C. Collagen fibers can be observed in both the 2PEM z-stack (top row panels, 2PEM) and the EM images of the serial sections (middle row, TEM). In the TEM images, the collagen is visible as electron dense fibers running in between the cellular material. Each column shows a 2PEM and an EM image obtained at a similar position in the sample, pre- and post EM processing. The numbers in the top left corner of the panels indicate their position in z to the image shown in the first column. The 2PEM dataset is stretched in z relative to the TEM dataset, causing the larger z-steps between the 2PEM images in the different rows. The bottom row shows the overlay of the 2PEM image (blue) and the TEM image (grey levels). Scale bars: 100 µm. D-F. The landmark-registration procedure is performed in Amira, using 3D models of collagen fibers as a reference. The selected landmarks (colored ‘targets’) indicate corresponding points in both datasets. D. 3D model of the collagen fibers (blue) and the cell of interest (green), obtained from a high magnification 2PEM z-stack of the cell of interest. E: 3D model of the collagen fibers (grey), obtained from a z-stack of EM images. F. The 3D visualization software Amira was used to overlay the landmarks and perform non-linear transformations to the 2PEM model to warp it into the EM model. G-I: Landmark-registration between the 2PEM and EM datasets enables retrieval and electron tomography of the cell of interest (green). G. Following the registration of the 2PEM in the EM dataset, the cell of interest could be docked within the z-stack of EM images. Scale bar: 10 µm. H. The docked cell of interest is outlined in green on a TEM section of the ROI. The nucleus of the cell is visible. Scale bar: 3 µm. K. Virtual slice through the tomogram obtained in the area that is boxed in I. The collagen fibers in the top left corner are clearly recognizable owing to their striated structure and high electron density. Scale bar: 500 nm. See Movie S4.

8 week old, female immuno-deficient mice (Rj:NMRI-Foxn1nu/Foxn1nu, Janvier labs) were transplanted with fluorescent tumor cells. For each experiment, a single mouse was subcutaneously injected into the ear with MDA-MB-231 cells expressing a cytoplasmic GFP [34] (∼15000 cells/µL, Figures 1, 2, 3) or D2.0R cells expressing LifeAct-Ypet [35]–[38] (5000 cells/µL, Figures 4, 5) in a 1∶1 mixture of phosphate buffered saline (PBS) and Matrigel (BD Biosciences). 8 mice were imaged 2–3 days following injection with MDA-MB-231 cells. 3 mice, injected with D2.0R cells, were imaged after 7 days, upon tumor growth. In general, shortly before intravital imaging, the mouse was first anesthetized through intra-peritoneal injection of a mixture of ketamine (100 mg/kg) and xylazine (10 mg/kg). Then, to visualize the blood vessels with 2PEM, 100 µL of Evans Blue (10 mg/mL in PBS, E-2129 Sigma-Aldrich) was administered to the anesthetized mouse via retro-orbital injection. A gas anesthesia (isoflurane) system was used for performing time-lapse imaging shown in figures 4 and 5. The mouse studies were performed according to the Guide for Care and Use of Laboratory Animals (E67-6-482-21) and the European Directive with approval of the regional ethical committee (CREMEAS for Comité Régional d'Ethique en Matière d'Expérimentation Animale de Strasbourg, AL/73/80/02/13). Mice received food and water ad libitum, they were checked daily and tumor growth never exceeded a week, leading to low-size tumors with no impact on the animal's health. All efforts were made to minimize suffering and euthanasia was performed using CO_2_.

### Intravital Two-Photon Excitation Microscopy (2PEM) and Near-Infrared Branding (NIRB)

The anesthetized mouse was mounted on a custom built holder inspired by Li *et al*. [29]. The holder keeps the ear flat under the lens of the microscope without interfering with second harmonic generation signal captured by a trans detector. The stage bearing the mouse was mounted on an upright confocal microscope (TCS SP8, Leica Microsystems, operating under Leica Application Suite Advanced Fluorescence (LAS AF) software), in an environmental chamber set to 28°C. 2PEM was performed by a single excitation wavelength at 940 nm (Chameleon Ultra II, Coherent), using a 25×0.95 N.A. water immersion objective. Emission wavelengths of 510 nm and 680 nm were chosen to collect the fluorescent signal of Life-Act (tumor cells) and Evans Blue (blood vessels), respectively, and the signal was collected by hybrid non-descanned photo-detectors (HyD, Leica Microsystems). The second harmonic generation (SHG, collagen) was imaged at a wavelength of 470 nm, using a photomultiplier detector (PMT, Leica Microsystems). The 2PEM datasets were captured at a scan speed of 400 Hz.

The ROI was marked by NIRB [12], [13], as described in the text. The frame contour was scanned with a pulsed laser at 800 nm (Coherent Chameleon Ultra II, coherent mean power 3,5W at 800 nm), and at a low scan speed (10 Hz, line average: 2, frame average: 2 and picture dimensions: 1024×1024 pixels) at the level of the skin surface, above the ROI. The branding was repeated until the square was clearly visible with transmitted light.

### Sample Preparation for Electron Microscopy

Following 2PEM, the ear was dissected out and chemically fixed for 1 hour at room temperature in 2.5% glutaraldehyde (GA, Electron Microcopy Sciences) and 2% formaldehyde (FA, Electron Microcopy Sciences) in 0.1 M sodium cacodylate buffer. Fluorescence microscopy images were collected to map the position of the NIRB markings on the ear skin, and the sample was stored overnight in fixative, at 4°C. The following day, the fixative solution was replaced by cacodylate buffer and the sample was kept at 4°C until further processing. The ear was then trimmed close to the ROI. For the samples injected with MDA-MB-231 cells, post-fixation was performed in 0.05% malachite green oxalate (Sigma) and 2.5% GA (Electron Microcopy Sciences) in cacodylate buffer for 2 hours on ice, followed by 0.8% K_3_Fe(CN)_6_ (Merck) and 1.0% OsO4 (Electron Microscopy Sciences) in cacodylate buffer, for 1 hour on ice. Note that malachite green is used here to reduce the lipid extraction commonly associated to sample processing in EM ([39] and references cited, [40]). As a result, the osmium tetroxide has more substrate to interact with which leads to an improved contrast of cellular membranes. Following each fixation step, the samples were rinsed 4×5 min with cacodylate buffer. The samples were then stained with 1% aqueous tannic acid (Electron Microscopy Sciences), for 1 hour on ice. Following 3×5 min rinses with sodium cacodylate buffer, the samples were washed 5×5 min in water and stained with 0.5% aqueous uranyl acetate (UA, Serva), for 1 hour at room temperature. After 5×5 min washes in water, the samples were dehydrated in acetone, infiltrated with a graded series of Epon (Serva) and flat embedded in a 400 µm thick layer of epon as described before [15]. Following overnight polymerization at 60°, the thin sample was cut out and mounted on an empty resin block. The sample was oriented such that the side with the NIRB markings was exposed, and the backside of the sample was glued to the block using a drop of epon. The sample was then again polymerized in the oven overnight. In this way, the sample was mounted so that the ultramicrotomy sections would be parallel to the imaging plane.

A similar processing protocol was used for the sample injected with the D2.0R cells, however the processing after primary fixation with FA and GA was performed in a PELCO BioWave Pro microwave processor (Ted Pella, Inc.). The malachite green/GA and K_3_Fe(CN)_6_/OsO_4_ fixation steps were done in the microwave, under vacuum, while exposing the sample to 14 minutes of 2 min ‘power on/off’-cycles (100 W). Rinsing in between the steps was partly microwave-assisted: the first rinses were performed in the hood, the final 2 rinses for 40 seconds at 250 W. The samples were stained with aqueous UA for 7×1 minute ‘power on/off’-cycles (150 W), under vacuum. The sample was then gradually dehydrated in 40 second steps of 25%, 50%, 75%, 90%, and 95% EtOH in water, at 250 W and at atmospheric pressure. Finally, the sample was rinsed twice in 100% EtOH (same settings). The sample was infiltrated with Epon by performing 7 steps (3 minutes at 250 W) where the concentration was increased from 25% Epon in EtOH to the 100% Epon, that was used for the last two washing-steps. The samples were then polymerized as described above. The microwave-assisted processing is advantageous over the bench-protocols since it reduces the time required for the procedure from days to hours. More importantly, it is believed that the microwave improves the infiltration of the chemicals into the sample. Indeed, we previously observed improved ultrastructural quality in these and other samples (data not shown) when comparing bench and microwave processing [41]. Thick sections (500 nm) were mounted on Superfrost Plus glass slides (Fisherbrand) and stained with a mixture of 0.5% toluidine blue and 1% borax in water. Thin sections (60 nm, Figure 2E and F) and semi-thick sections for tomography (240 nm, Figure 3 and 5) were mounted on formvar-coated slot grids. These sections were stained for 5 minutes with 2% UA in 70% methanol and next for <3 min with lead citrate (Electron Microscopy Sciences).

### Electron Microscopy Imaging

The thin sections were imaged with a CM120 transmission electron microscope (TEM, FEI Company) operating at 120 kV. Images were obtained with a Keen View CCD camera (Soft Imaging Solutions, Olympus), running under iTEM (Olympus) software. Electron tomography was performed with a Tecnai F30 Field Emission Gun TEM (FEI Company) operating at 300 kV and equipped with an Eagle 4K camera (FEI Company). The F30 was controlled by Tecnai User Interface (PEOUI) and Tecnai Acquisition (TIA) software. Single-axis tomograms were obtained using SerialEM [42], and reconstructed in eTomo, part of the IMOD software package [43] (Boulder Laboratory, University of Colorado).

### Image Processing

Fluorescence images were processed in Fiji, part of ImageJ [44], IMARIS software (Bitplane), and in Adobe Photoshop CS6. Photoshop CS6 was also used to process the EM images. 3D registration of the serial sections and of the tomograms was performed in 3dmod, part of the IMOD software package [43] (Boulder Laboratory, University of Colorado), and the movies were produced in 3dmod, Fiji and Amira (FEI Company). Alignment, transformation and registration of the 3D models of the 2PEM and EM datasets were performed in Amira.

## Results

### Intravital imaging of tumor cells and branding the imaged area

In order to study *in vivo* tumor invasion events using intravital imaging, we employed a mouse model where fluorescent tumor cells were injected subcutaneously into the ear. The mouse ear was chosen as a model system since it enables non-invasive intravital microscopy through the skin. Moreover, the ear provides excellent imaging conditions for 2PEM since it is thin, easily accessible, and less prone to breathing-induced imaging artifacts. Figure 1A outlines the correlative workflow, the main steps of which are illustrated in panels B-G. After injection of the tumor cells, the mice were anesthetized and mounted on a custom-made stage (Figure 1B). The stage was fitted with a holder in which the ear with the grafted cells was stabilized [29]. Figure 1C depicts a z-projection from a representative 2PEM stack (Movie S1), revealing fluorescent tumor cells in green (GFP), collagen fibers in blue (SHG) and the blood vessels in red (Evans Blue). Following 3D imaging, the position of the imaged area was marked at the skin level, above and sufficiently distant from the imaged cells to avoid any NIRB-induced damage [45], [46] (Figure 1D). Hereto, the contour of a square frame was slowly line-scanned using a high power pulsed laser. NIRB resulted in auto-fluorescent physical markings that could be seen upon imaging the same area after branding (Figure 1E, Movie S1). By performing NIRB on the living tissue, immediately after 2PEM imaging, the markings can be quickly and accurately created by simply changing the focus of the microscope to the level of the skin surface. Performing the NIRB post-fixation, as described by others [45], [46], would require a transfer of the mouse from the stage of the microscope to the bench for fixation and therefore jeopardize the tracking of the ROI position.

After chemical fixation, a low magnification fluorescence image was obtained to map the position of auto-fluorescent NIRB markings in the ear sample (Figure 1F). Based on this FM map, a smaller sample containing the imaged volume was produced and prepared for EM. Since the skin layer of the mouse ear significantly hinders the infiltration of the chemicals, we have overcome this issue by cutting down (using the NIRB mark) the sample as close as possible to the ROI. In the resin block, the sample was oriented so that subsequent sectioning could be performed parallel to the 2PEM-imaging plane (Figure 1G). The ROI was approached by producing serial sections from the resin embedded sample. Serial thick sections (500 nm), mounted on glass slides and stained with toluidine blue, can be inspected by light microscopy (LM). These images offer a quick overview of the structural features in the sample. Thin sections are mounted on slot grids and are imaged at high resolution by TEM (Figure 1G).

### Sequential LM/EM to Retrieve the Cell of Interest

Although the NIRB markings outline the area of the sample that contains the ROI, they do not reveal how deep within the tissue the cells of interest can be found. To retrieve the tumor cells, it is therefore necessary to carefully monitor the progression to the ROI while serial sectioning through the sample. As an initial method to correlate between 2PEM and EM, we chose to use a classic approach for the correlation [47], consisting in alternating between semi-thin (500 nm) and ultra-thin (60 nm) sections of the area of interest. LM images of the semi-thin sections are used to visualize the global anatomy of the tissue, which can then be compared to the 2PEM dataset. When the cells of interest are localized on the semi-thin sections, they can be easily retrieved in the consecutive thin sections and imaged by EM.

Figure 2A and B show respectively a single plane and a 3D representation of a 2PEM z-stack of mouse ear tissue injected with tumor cells, which express cytoplasmic GFP (Movie S2). As a target for EM imaging, we selected a single cell that had an apparent polarity and invasive morphology, with one side pointing towards the blood vessel (Figure 2A and B, square). Following NIRB, the sample was processed for EM (Figure 1, Methods). The 2PEM dataset revealed that the cell would be found>50 µm (data not shown) below the skin surface, giving an approximation of the depth of tissue to be trimmed. The ROI was first approached by producing 500 nm serial sections, which were imaged with LM (Figure 1G and Figure 2C, ‘Approach + Correlation’). LM of the sections revealed the NIRB markings (data not shown) and other structural features of the tissue, including blood vessels. These features were also notable in the 2PEM dataset and could thus be used as reference points for correlating the volume acquired via 2PEM with the one from the resin embedded sample. While closely monitoring the progression through the sample, the vessels visible in the 2PEM stack (Figure 2A) were identified in the thick sections. From the 2PEM z-stack we could predict that our cell of interest should appear approximately 3.5 µm below this first set of serial sections. We then collected a sequence of thick and thin sections of the area of interest (Figure 2C). Overview images of the LM sections were correlated to the 2PEM z-stack (Figure 2A, Figure 2C, ‘Correlation ROI’, and D). Finally, the cell of interest was identified based on its shape and its position relative to one of the vessels (Figure 2A and 2D, arrowhead). Subsequently, this same cell was retrieved in the consecutive series of 60 nm sections (Figure 2C, ‘EM Imaging ROI’) and imaged at high resolution with EM (Figure 2E and F, Movie S2) revealing ultrastructural details of its protrusive region.

### Serial EM of Invading Tumor Cells

The procedure described above (Figure 2C) is an efficient way to correlate FM and EM. However, it does not allow full three-dimensional reconstruction of the tumor cells with EM since it dedicates part of the volume of interest to sections for LM. In order to enable EM imaging of the complete ROI, it is necessary to obtain serial EM sections of the full volume. In this second approach we focused on tumor cells that were in close proximity to a large blood vessel (Figure 3A and Movie S3). For a better understanding of their distribution within the selected volume, the 2PEM dataset was segmented and the cells of interest rendered in green and numbered (Figure 3B). Orthogonal views (XZ) of the 2PEM dataset (Figure 3C) reveal the position of the cells of interest (circled) relative to the vessels (red, Evans Blue) and to the collagen-rich skin surface (blue, SHG). To approach the ROI (Figure 3D, ‘Approach + Correlation’), serial thick sections were imaged by LM and aligned. The image stack was then segmented (Figure 3E and F, Movie S3) to highlight the NIRB marks (purple), hair follicles (orange) and vessels (red) using 3dmod [43]. The resulting 3D model of the sample features (Figure 3F) was then correlated to the model from the 2PEM dataset. We could monitor the block-face position within the sample by overlaying the two 3D models (Figure 3G and Movie S3). The three vessel bifurcations that could also be recognized in the 2PEM dataset (Figure 3G, asterisks, and Figure S1A, V1-3) were key features to try to predict the position of the cells of interest. Three of the cells (3, 5 and 6) are in close proximity to these vessels, but slightly deeper into the tissue (about 1 to 2 µm as estimated from the measurements done on the 2PEM data-set). Having approached the cells of interest, we switched to producing serial EM sections (Figure 3D, ‘EM imaging ROI’).

Based on the 2PEM dataset, we could anticipate the cells of interest being within 50 µm in depth below the mentioned vessel bifurcations (in the z-range 73–123 µm of the imaged volume, Movie S3). To ensure that we could image all selected cells with EM, we obtained>170 serial sections, approximately 300 nm thick that were suitable for EM tomography. This ‘library’ of sections represented the complete volume in which the cells of interest could be expected. By aligning low magnification TEM images of these sections to the 2PEM z-stack using Photoshop, the xy positions of the cells could be determined. Using these coordinates as a guide, we could retrieve the selected tumor cells (Figure 3H, 1–6). The correlation was further confirmed by the shape similarity of the cells and their position relative to the vessel. Serial sectioning of the sample enabled us to reconstruct 3D EM models of the cells of interest through segmentation of the cells' plasma membrane (Figure 3I-K). This provided ultrastructural information of the tumor cells, and further confirmed the correlation of the two datasets (compare Figure 3I and K). Importantly, the gain of resolution between 2PEM and EM can be appreciated even though EM is performed here at low magnification (Figure 3I-K). For example, while the cell appears to be very close to the blood vessel in the 2PEM dataset (Figure 3I), serial EM reveals that it is actually still physically separated at least 5.5 µm, from the lumen of the vessel by both cellular and a-cellular material (Figure 3J and K).

Further gain in resolution can be achieved by electron tomography, which enables 3D visualization at nanoscale resolution. We selected a highly-polarized cell with protrusion-like extensions in close proximity to fibrous material, very likely extracellular matrix (ECM) fibers such as collagen (Figure 3L). A virtual slice from a tomogram, obtained at the site of these protrusions, shows the complex organization of the cell membrane in close proximity to the ECM fibers (Figure 3M, Movie S3). The latter are sufficiently electron dense to allow for semi-automatic segmentation throughout the reconstructed 3D volume (Figure 3N and Movie S3, blue). Altogether, this proves that we can potentially target any event/cell of interest within the processed ROI and image/segment it at high resolution in 3D.

Identification of corresponding cells and other structural features (vessel branches) in both datasets allowed producing simplified 3D maps of the 2PEM z-stack and the serial section dataset. For both datasets, the relative positions of structural features, i.e. cells and vessel branches (Figure S1A), were plotted within a 3D coordinate system. In the 3D Analysis Software Amira, it was possible to simultaneously visualize both maps in 3D and to select common features, ‘landmarks’, in both datasets (Figure S1B). Together, the coordinates of the selected points form two paired ‘landmark-sets’, which are the basis of the correlation between the two datasets. Using Amira's *LandmarkWarp* module, we performed a transformation of one map to fit into the other by attempting to overlay the paired landmarks in 3D. Applying a ‘rigid’ transformation method will optimize the warp without any deformations, but solely by translation and rotation of the dataset. The ‘Bookstein’ transformation method does allow additional non-linear transformations of the model, including stretching and scaling, in order to make both landmarks-sets overlap. Comparing the two different transformation approaches to fit the 2PEM in the serial sections model reveals the differences between both datasets. Using rigid transformations, it was not possible to overlay the landmarks in 3D (Figure S1C); this could only be achieved by the non-linear transformation enabled by the Bookstein method (Figure S1D). This finding confirms that, as expected, non-uniform changes are introduced while processing living tissues for EM. Therefore, the position of the cells of interest in the 2PEM dataset cannot be directly translated to their location into the resin block and therefore in the stack of serial sections. This further suggests the need for reliable correlation methods to allow the easy retrieval of events of interest in the EM processed sample.

### Time-lapse imaging of invading tumor cells

One of the major advantages of intravital imaging is to follow tumor development over time. Here we show how to combine time-lapse 2PEM of fully mature tumors with 3D EM. D2.0R cells [38], expressing the fluorescent actin marker LifeAct-YPet [35], [36], were injected into the mouse ear tissue. We obtained fully-grown, invasive and vascularized tumors within 14 days after injection (Figure 4A, top left panel). While tumor size made 2PEM imaging more challenging, we could still analyze invasive regions at the tumor borders and record isolated invading cells (Figure 4B and C, Movie S4). Usage of LifeAct-Ypet allowed to dynamically record, through time-lapse imaging of the anesthetized mouse, invasive protrusions that resembled the filopodia-like protrusions (FLPs) previously characterized *in vitro* [35], [36] (Figure 4E, Movie S4). While the time-lapse acquisition of actin reveals how FLPs probe the surrounding environment, recording of SHG and the Evans Blue label allows to respectively visualize the collagen meshwork that engulf the imaged cell as well as the local hemodynamics (Movie S4). Following 2PEM of this cell, the imaged area was marked at the level of the skin surface using NIRB.

### Using the collagen fibers meshwork to retrieve the ROI

In the previous experiments, the vasculature was used to retrace the position of the cells of interest within the tissue. In the case of mature tumors, the tumor-associated vasculature can be perturbed and therefore difficult to use as fiducial landmarks. Moreover, a mature tumor such as the one shown in figure 4 is enriched in fibrous and dense cellular material in which the cancer cells are evolving (Figure 4A). As a consequence, the strategy for targeting cannot rely on a 3D mapping by LM from semi-thin sections as shown previously. In the situation illustrated in Figures 4 and 5, the best strategy is therefore to perform a minimal approach of the region of interest by semi-thin sections (500 nm), that would expose the NIRB markings only, and then to collect the rest of the tissue by serial sections for TEM (Figure 5A).

Using this approach we obtained a complete 3D TEM dataset of the ROI and its surroundings, which enabled full volume correlation between 2PEM and TEM. From both the 2PEM and TEM dataset we could build precise 3D maps of the tissue, and the landmarks therein. Having identified corresponding landmarks, we could overlay the two datasets through 3D registration. A simplified cartoon of the steps involved in this approach is shown in Figure 5B. As revealed by non-linear 2PEM (SHG) and TEM, the collagen fibers form dense bundles within the tissue (Figure 5C). They are distributed as an organized meshwork that can be used as landmarks in both modalities. As the 2PEM dataset also provides the position of the cell of interest relative to the collagen fibers (Figure 5B, dashed lines), the registration transfers the predicted position of the cancer cell within the EM dataset. Using Amira, the collagen fibers were modeled from the 2PEM dataset and from the EM z-stacks (Figure 5D and E). Additionally, we also segmented the cell of interest in the 2PEM dataset (Figure 5D). Visualizing both 3D models in a shared 3D space in Amira enabled to identify corresponding points in both datasets, e.g. cross-points between collagen fibers. These similarities in both datasets could be selected with ‘*Landmark (2 sets)*’. Using Amira's *Landmarkwarp* module, the 2PEM collagen model with the ROI was then warped into the EM collagen model. This required non-linear transformations of the 2PEM model (Figure 5F). This finding is in agreement with our efforts to align a 2PEM and serial section 3D maps of structural reference points (Figure S1). Here, it was not possible to overlay the corresponding points from the pre- and post-EM-processing maps without performing non-linear transformations.

In Amira's virtual 3D space, the collagen models were linked to the image z-stacks from which they are segmented. Warping the 2PEM model to fit within the TEM model therefore also results in the projection of the ROI on the series of TEM images from the serial sections (Figure 5G). Having established the approximate position of the cell within the series of serial sections, high-resolution images could be made in this area (Figure 5H). Electron tomography of a selected area (Figure 5J, dashed square, Movie S4) enables 3D EM imaging of the cell of interest. Moreover, a virtual section of the tomogram (Figure 5I, Movie S4) has an improved z-resolution over the 2D projection of the full section, which is normally obtained with TEM. Here, the striations of the collagen fibrils, close to the cell membrane, are clearly visible, and the structure of the cancer cell can be studied in detail.

## Discussion

Searching for individual cells of interest in complex tissues embedded in a resin block is a tedious task. In light microscopy, cells or regions of interest are routinely tagged with fluorophores. Targeting to their specific location is thus fast and accurate even in complex organisms, provided that light microscopy allows imaging deep within intact tissues. To perform similar targeting in electron microscopy, several attempts have been made to preserve the signal from fluorescent proteins [48] or to add fluorescent dyes to the tissues [49] in resin embedded samples. These techniques rely on specific fixation methods (cryo-methods) and also on the use of hydrophilic resins (methacrylates). When the use of fluorescent probes is not possible, or when the selected procedures to prepare the sample for EM lead to a quenching of the fluorescent signal [8], other strategies need to be used [12], [13], [15], [16], [50], [51]. In this paper, we combine and synergize previously published strategies in order, for the first time, to perform accurate and reliable correlative light and electron microscopy of tumor cells in vivo upon non-invasive 2PEM imaging. One consists in recording the volume of the living sample to build a three-dimensional map of the region of interest [15], [52], which is used to predict its position in the resin block. Another relies on artificial landmarks produced with a near-infrared laser to highlight the region of interest directly on the hydrated sample [12]–[14]. Finally, we improved a strategy that we used before [15], [52] consisting of recapitulating the local anatomy of the tissue with serial sections in order to retrace the position of individual objects within the EM dataset [16].

Our method of correlation has three variations. With the first, sequences of thick and thin sections enable quick screening of the sample for the ROI, by comparing the 2PEM model to the 3D reconstruction built from the LM acquisitions of serial thick sections. Upon identification of the tumor cell in these sections, its ultrastructure is recorded by TEM from the adjacent thin section (Figure 2). A disadvantage of this approach is that it allocates a significant fraction of the ROI to the model reconstruction by serial LM imaging. Although these thick sections could in principle be re-embedded and re-sectioned for EM, full-cell reconstruction will not be possible and crucial structures may be lost. With the second strategy, a fraction of the embedded tissue is used to create a partial 3D model, which is correlated to the 2PEM dataset of the same area. The major advantage is the preservation of the cells of interest for further TEM imaging. Moreover, it facilitates an accurate estimation of the depth at which cells should be expected within the resin block. Full volume correlation allows determining the position of the ROI (Figure 5B). Using the 3D imaging software Amira [53], the registration of the two datasets required non-linear transformations of the 2PEM model to fit into the model obtained from the serial TEM images (Figure S1) reflecting the sample distortions caused by the preparation methods [54]. Chemical fixation, dehydration and resin embedding each influence the volume orientation of the sample. During chemical fixation, GA will cause around 5% shrinkage of the sample [55], whereas OsO_4_ fixation will result in swelling [54], [56], [57]. Subsequent dehydration and resin embedding will cause further shrinkage to the specimen, which can be minimized by exposing the sample as briefly as possible to the solvents [55], e.g. by microwave assisted embedding. Importantly, the sample distortions are never homogenous throughout the tissue due to differences in biological material and infiltration efficiency of the chemicals. This explains why the 2PEM dataset does not linearly corresponds to the TEM dataset. As a consequence, the prediction of the position of one particular structure inside the resin-embedded sample has a limited accuracy (in the order of 20 µm). Taking this into consideration, the progression through the sample has to be performed with great care and, upon approaching the ROI, thin sections for EM should be collected systematically. Similar concern is illustrated with the third approach, where the cells of interest are expected close to the surface of the sample (within the first 10 microns of the resin block). In that case and for preserving the cells of interest, systematic serial sections are collected and recorded by TEM. The correlation is then performed *a posteriori* by overlapping the 3D dataset from both imaging modalities. In this work, we show that endogenous landmarks can serve as fiducial for the volume registration. We have used blood vessels bifurcations as well as fluorescent cancer cells to build sets of points that were paired and matched together in Amira. The pairing can only be performed if the landmarks are clearly visible in both imaging modalities. Their detection is made possible in fluorescence microscopy by the use of dyes or of fluorescent proteins, while in EM, they show a straight forward pattern that can be identified unambiguously. In some instances though, tissues can be too dense or be perturbed by the onset of a large and vascularized tumor, making the vessels recognition difficult in both FM and EM. As illustrated in figure 5, in such conditions, the density of the tissue as observed by TEM hinders significantly the recognition of the targeted cell. Nevertheless, our approach was versatile enough to allow the use of other structures for the correlation, such as the collagen meshwork, which is again visible in both imaging modalities. Indeed, collagen fibers can be detected from living tissues using SHG, and appear as bundles of electron dense fibers in EM micrographs. The segmentation of these fibers on both datasets allowed a precise registration (after non-linear transformations) and a prediction of the position of the cell of interest.

The use of anatomical features as a basis for correlation can also be applied to studies of other biological questions in a wide range of samples and applications, making it a highly versatile approach. We have demonstrated correlation between FM, LM and EM based on different examples of intrinsic fiducials. Moreover, the approach described here could be extended by recently developed, advanced 3D EM approaches, including Focused Ion Beam Scanning EM [58] and serial block-face electron microscopy [59]. In both methods, numerous cycles of sequentially imaging and removing the sample surface result in voluminous 3D EM datasets. Correlation of the serial-sectioning dataset and the 2PEM model enables identification of the ROI within the sample, which can then be imaged either of these techniques.

With these improved methodologies for correlating dataset from multiphoton microscopy to 3D electron microscopy, we bring an accessible set of solutions to perform structure-function analysis upon intravital imaging of tumor behavior in a murine model. In this paper, we show that mouse intravital imaging of a tumor can be efficiently correlated to the ultrastructural analysis of selected cancer cells. The subcutaneous graft model in the mouse ear has several advantages for studying the behavior of cancer cells in a native microenvironment, as it allows functional imaging over time, the capture of rare events at the single cell level. Moreover, our technique allows the recovery of the same cells for ultrastructural analysis. We therefore expect such methods will allow the study of fundamental features of the metastasis cascade within intact tissues.

## Supporting Information

Figure S1 **3D Registration of 2PEM and Serial Section Datasets.** A: Identification of features, 'landmarks', in the 2PEM dataset that could be retraced in the sequence of serial sections obtained from the EM processed sample. B. Front and side-view of 3D maps of the positions of the features in the 2PEM dataset (colored spheres) and the corresponding EM coordinates (colored squares). The vectors indicate for each EM position the magnitude (in µm) and the direction of the offset between the two datasets. In an attempt to overlay the 3D maps of both datasets, the 2PEM map (circles) was transformed to dock it in the serial-section dataset (squares). In Amira, it is possible to perform a 'rigid transformation' (C), which lowered the offset between the corresponding dataset, or to force the paired 'landmarks' (features) to overlap by performing a 'Bookstein Transform' (D).
(TIF)

Checklist S1 (PDF)

Movie S1 **2PEM Imaging and NIRB.** GFP-expressing tumor cells (green) are subcutaneously injected and imaged with 2PEM. Vessels are stained with Evans Blue (red) and collagen fibers are visualized through SHG (blue). The region of interest is then marked by NIRB (orange).
(MP4)

Movie S2 **Zooming in to the cell of interest.** After injection with GFP-expressing tumor cells (green), the skin tissue was imaged with 2PEM. Vessels are shown in red. The z-stack moves into the tissue and then zooms in on a cell of interest. Following EM processing, thick and thin sections of the sample were correlated to the 2PEM z-stack, and the tumor cell was imaged at high magnification.
(MP4)

Movie S3 **Full Volume Correlation of the ROI Enables Electron Tomography of Tumor Cells** ***in vivo*** **.** Mouse skin tissue injected with GFP-expressing tumor cells is imaged with 2PEM. Vessels are shown in red (Evans Blue dye) and collagen (SHG) is shown in blue. 500 nm sections through the EM processed sample are aligned segmented. The 3D models of the 2PEM and the serial section datasets are correlated to monitor the approach to the cells of interest. Upon retrieval of the cell of interest, electron tomography reveals its ultrastructure in 3D. Scale bar: 500 nm.
(MP4)

Movie S4 **Correlating Intravital Time-Lapse 2PEM to Electron Tomography of D2.0R Tumor Cells.** The 2PEM z-stack reveals the position of LifeAct-Ypet expressing D2.0R cells injected in mouse ear skin. The vessels were injected with Evans Blue (red) and the collagen is visible through SHG (blue). The cells of interest were imaged over a period of 120 minutes (one image was acquired every three minutes), capturing the formation of actin-rich (LifeAct-Ypet signal, green) protrusions. Correlating 2PEM to EM enables to zoom in and perform electron tomography to reveal the ultrastructure of the cell of interest and the collagen fibrils in the extracellular matrix. Scalebar: 500 nm.
(MP4)
