# Supplementary figures and images for: Correlating Intravital Multi-Photon Microscopy to 3D Electron Microscopy of Invading Tumor Cells Using Anatomical Reference Points
Source: PLoS One. 2014 Dec 5;9(12):e114448. doi: 10.1371/journal.pone.0114448 (PMC4257674; doi:10.1371/journal.pone.0114448)

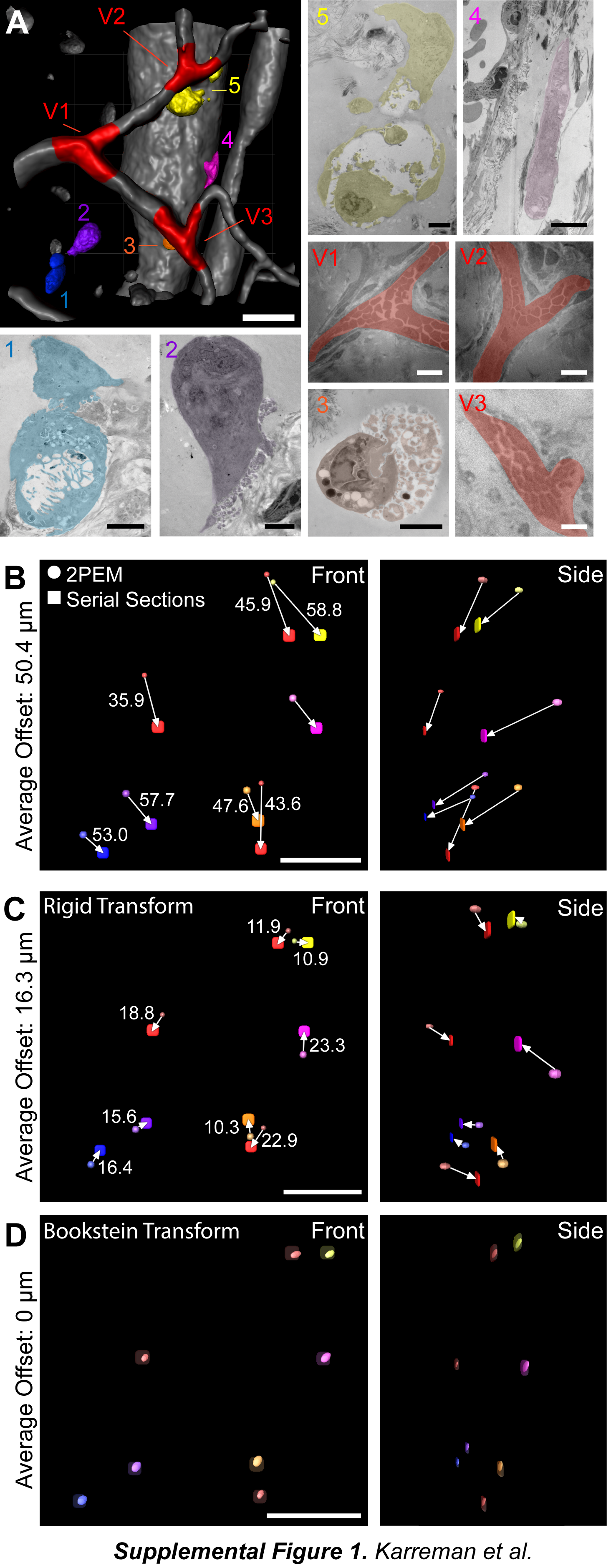

Supplement: Figure S1 — 3D Registration of 2PEM and Serial Section Datasets. A: Identification of features, 'landmarks', in the 2PEM dataset that could be retraced in the sequence of serial sections obtained from the EM processed sample. B. Front and side-view of 3D maps of the positions of the features in the 2PEM dataset (colored spheres) and the corresponding EM coordinates (colored squares). The vectors indicate for each EM position the magnitude (in µm) and the direction of the offset between the two datasets. In an attempt to overlay the 3D maps of both datasets, the 2PEM map (circles) was transformed to dock it in the serial-section dataset (squares). In Amira, it is possible to perform a 'rigid transformation' (C), which lowered the offset between the corresponding dataset, or to force the paired 'landmarks' (features) to overlap by performing a 'Bookstein Transform' (D). (TIF) [file pone.0114448.s001.tif]
